# Physical and morphological characterization of the 19 May 2021 ash cloud deposit at Stromboli (Italy)

**DOI:** 10.1038/s41598-022-14908-1

**Published:** 2022-06-24

**Authors:** Giuseppe Re, Massimo Pompilio, Paola Del Carlo, Alessio Di Roberto

**Affiliations:** Istituto Nazionale Di Geofisica E Vulcanologia, Sezione Di Pisa, via C. Battisti 53, 56125 Pisa, Italy

**Keywords:** Natural hazards, Volcanology

## Abstract

We report on the ash cloud related to the gravitational collapse of a portion of the Stromboli volcano crater rim that occurred on 19 May 2021. The collapse produced a pyroclastic density current (PDC) that spread along the northwest flank of the volcano and propagated in the sea for about 1 km. The PDC was associated with a convective ash cloud that rapidly dispersed eastward and deposited a thin layer (< 1 mm) of very fine pinkish ash over the village of Stromboli. The deposit was sampled shortly after the emplacement (within a few hours) and prior to any significant reworking or re-sedimentation. We present a comprehensive description of the deposit including dispersal, sedimentological characteristics and textural and geochemical features. We also compare the 19 May 2021 deposit with fine-ash deposits connected to other PDCs and landslides previously occurring at Stromboli and with the distal ash of a paroxysmal explosive eruption of Mt. Etna volcano. Results indicate that the distributions of the mass on the ground and of the grain size are not correlated with the distance from the source. Also, the componentry reflects a preponderance of remobilized material ingested by the PDC. Therefore, the great amount of fine ash can be ascribed to clasts comminution processes, although the prevalence of dense crystalline components records an overall equiaxial shape, revealing a paucity of elongated clast with complex morphology. Furthermore, the outcomes of this work aim to create a collection of data of a co-PDC ash cloud that may prove useful for comparison with other deposits worldwide.

## Introduction

Pyroclastic density currents (PDCs) are typically generated during eruptions fed by andesite to rhyolite magmas, as a result of eruptive column collapses (e.g., Mt. Vesuvius, 79 AD, Italy^1^), lava dome collapses (e.g., Soufriere Hills, 1995, Montserrat^2,3^), or laterally directed explosive blasts (e.g., Mount St- Helens, 1980, USA^4^). PDCs have also been witnessed at volcanoes fed by mafic magmas as a hybrid phenomenon between a landslide and a PDC, with triggering mechanisms mainly connected to the collapse of the crater rim^5–9^ or the remobilization of hot pyroclastic materials deposited on steep slopes during the explosive activity^10–14^. In these cases, PDCs have small volume (10^4^–10^7^ m^3^), relatively high temperatures (360–700 °C), but can travel some kilometers from their source^8,11,12,15–17^, posing severe threats to anyone in the vicinity of the volcanoes. PDC can be accompanied by clouds of extremely fine elutriated ash (co-PDC plumes) that often deposit a very thin (in the order of millimetres) ash blanket, which generally very short-lived. Stromboli is one of the volcanoes where this type of phenomena has been most frequently observed, with at least four events in the last ten years (12 January 2013; 6–7 August 2014; 31 March 2020, 19 May 2021), and volumes involved in the collapses in the order of 10^4^–10^5^ m^3^.

**Figure 1:**
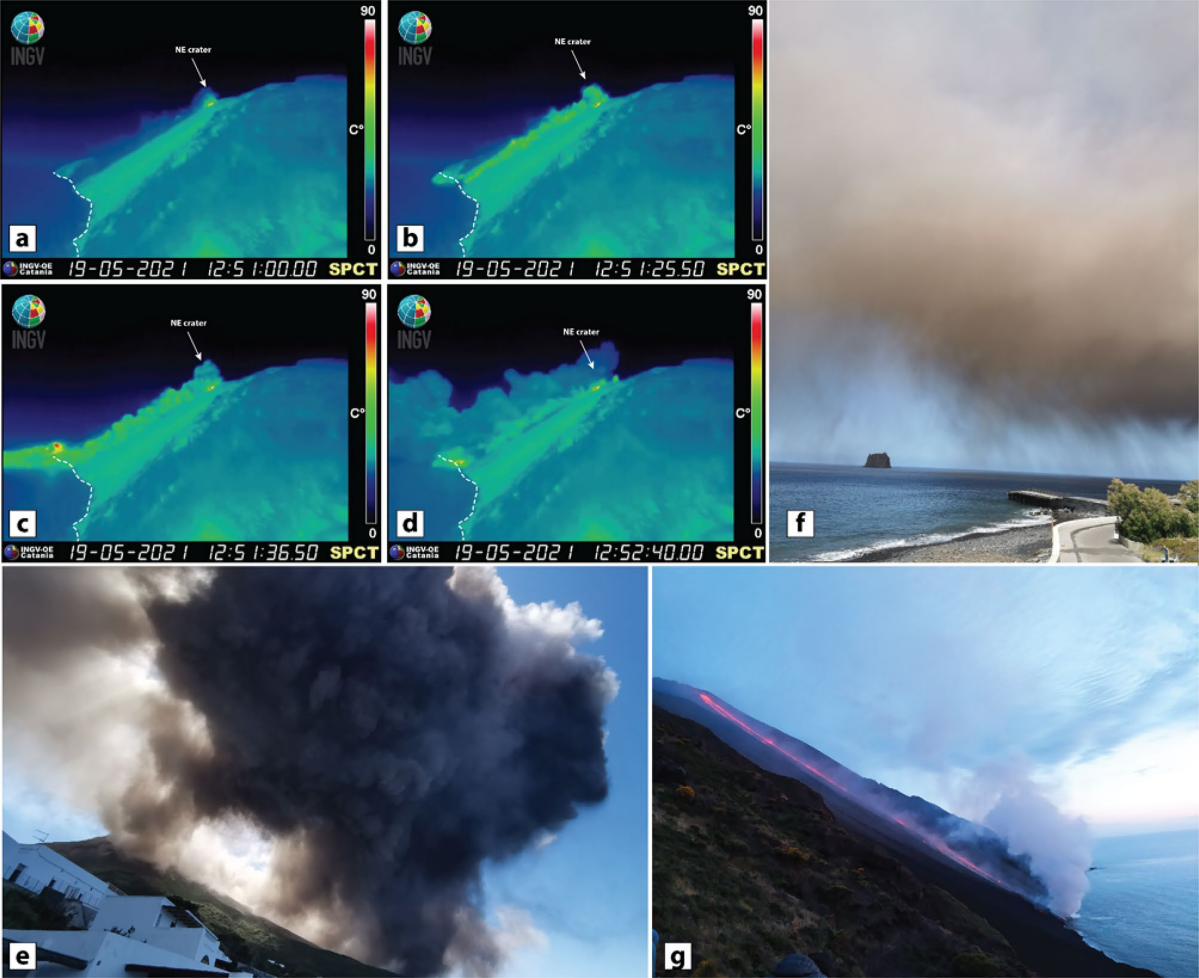
Photos (**a**),(**b**),(**c**) and (**d**) illustrate successive moments of the collapse and related PDC emplacement from the INGV thermal camera located at Punta dei Corvi, the SW side of the Sciara del Fuoco (SdF); the arrow indicates the NE craters, and the white dashed line indicates the shoreline. (**a**) represents the early stage of the collapse, and the cold dust is visible above the SdF, lifted up by the continuous rock falls lasting a few hours. (**b**) the PDC is visible running down the SdF, propagating offshore above the seawater table (**c**), and a small accretion fan besides the shoreline. The interaction with the warm PDC and the cold marine water caused hydromagmatic explosions, with flares visible in (**c**). In (**d**) the rising ash-cloud can be seen; there is a portion along the SdF that is elutriated from the PDC, and a portion offshore produced by the hydromagmatic explosion triggered after the PDC water interaction (see the high temperature burst in **c**). (**e**), taken from Ficogrande, shows the ash cloud rising above the village of Stromboli; it seems that the most voluminous portion of the cloud sources offshore. Photo (**f**) captures the ash fingers settling from the cloud. Photo (**g**) details the lava pouring out from the breached crater rim. Hydromagmatic explosions occured once the lava entered the sea, and a column of steam raised.

In 2021, from the beginning of May, a progressive increase in the frequency and intensity of the Strombolian activity was observed by the monitoring network and direct observations (see INGV-OE Reports of 04, 11 and 18 May 2021^18–20^). From 11 May 2021, intense and irregular spattering episodes occurred at the volcano summit, predominantly at the northern crater N2, building up a small scoria cone^21^. On 19 May 2021 at 12.51 UTC, a portion of the northern sector of the crater collapsed (Fig. 1a) producing a small volume PDC that propagated downslope, within the Sciara del Fuoco (SdF), at a speed of c. 50 km/h (Fig. 1b) and over the sea for about 1 km (Fig. 1c). The PDC, comprising a volume of material estimated between 10,000 and 70,000 m^3^^22,23^, produced a tsunami wave of c. 20 cm on entering the sea. The collapse occurred without any explosion quake and infrasonic signals, indicating that it was not associated with any major or paroxysmal explosion. The tiltmeters array, located at the volcano summit, recorded a deflation signal of ~ 1.8 µrad associated with the collapse^23^.

**Figure 2:**
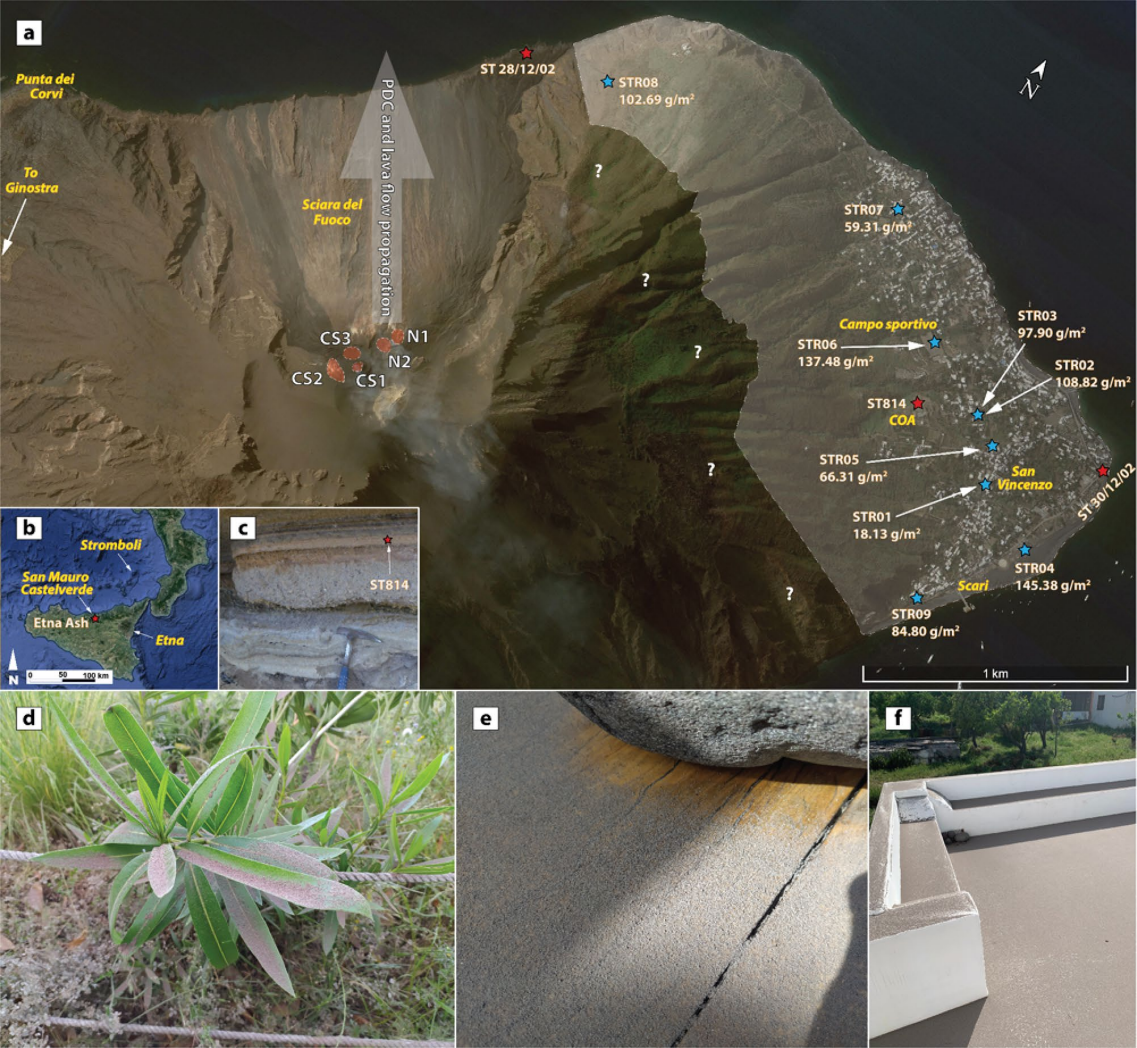
(**a**) Map of the northern portion of Stromboli island (Google Earth@2022 Map data SIO, NOAA, U.S.Navy, NGA, GEBCO). White-dashed red-filled shapes indicate the summit craters (N1, N2—Northeast craters; CS1, CS2, CS3—Central-South craters). The shaded arrow approximately indicates the propagation direction of the PDC and lava flow. The blue stars in the map indicate the sampling locations of the 19 May 2021 ash cloud deposit, and for each sample is annotated the mass value (g/m^2^). The red stars indicate the sampling locations of three samples used as comparison, for instance the 28 December 2002 (ST281202), the 30 December 2002 (ST301202) and the COA ash tuff (ST814). The white shaded area delimits the ash fall dispersion (question marks indicate that it was not possible to verify whether the ash cloud deposit occurred at higher elevations). Toponyms are in yellow and italics. The inset picture (**b**) illustrates a regional view of Sicily, and the red star marks the sampling location of 22 February 2021 Etna Ash (Google Earth@2022 Map data Landsat/Copernicus, SIO, NOAA, U.S.Navy, NGA, GEBCO). The inset picture (**c**) depicts the COA pyroclastic sequence, and the stratigraphic position of ST814 is annotated. Photos (**d**),(**e**) and (**f**) detail the tephra deposit investigated in this paper. In particular, picture (**f**) corresponds to the location of samples STR02 and STR03.

As soon as the PDC propagated in the SdF, its top began to entrain the air, decreasing the bulk density of the part of the current involved, causing a density stratification. As part of the interaction between the PDC and the overlying air, a mixture of heated air, volcanic gas and fine-grained volcanic fragments were elutriated upward from the main body of the current. Images from the thermal camera did not register high temperature for the PDC (Fig. 1b), although when it entered the sea, it caused hydromagmatic explosions, suggesting a relatively high temperature of the main PDC body (Fig. 1c). A convective cloud (Fig. 1d, e and movie in Supplementary Video 1) of steam and ash was generated by the combination of these two processes. The ash cloud swelled in a thick grey plume (Fig. 1e) that rose upward and carried by the wind propagated eastward (Fig. 1f), blanketing the village of Stromboli with a very thin layer of pink fine ash (Fig. 2a). Eyewitnesses revealed that the northern and eastern parts of the island were completely shrouded in the cloud, while the southern and western parts were free from falling ash.

The collapse of the crater terrace rim and the breach of the uppermost part of the conduit also facilitated the overflow of magma into the SdF (Fig. 1g), which produced a lava flow (at 13.08 UTC) that rapidly reached the shoreline and was active for a few days^22,23^.

We sampled the 19 May ash cloud deposit just a few hours after the deposition, which proved an invaluable opportunity to investigate the deposit before any significant remobilization or reworking. Generally, such kinds of deposits are ephemeral and susceptible to easy removal/reworking by atmospheric agents (e.g., wind, rain, etc.), or anthropic activities (e.g., cleaning of the urban areas), consequently are rarely preserved in the stratigraphic records. This paper aims to describe the ash cloud deposit by means of field distribution, lithological and sedimentological characteristics, and textural and geochemical features. We also compared the studied deposit with four other fine-ash samples. Three of them were collected at Stromboli, including the ash samples related to the 28 December PDC^24^ and 30 December landslide^25^ occurring during the 2002 eruption, and the pink ash tuff sampled at the top of the COA (Advanced Operative Centre) pyroclastic succession (Fig. 2). The fourth sample was from the distal ash fallout related to the 22 February 2021 Etna paroxysm, which settled about 75 km NW from the vent. This comparison aims to evaluate similarities with products related to analogue processes in terms of grain size distributions (GSD), componentry and clast morphometry. Results of the characterization of the physical properties of ash particles provide important data for understanding fragmentation (generation) processes, transport and deposition, and for the fluid-dynamics modelling of volcanic plumes, useful for dispersal purposes^26^.

## Results

### Mass of the deposits and grain size distributions

The 19 May 2021 ash cloud deposit consists of a < 1 mm thick continuous layer of very fine (dusty) ash, pinkish to purplish in colour, that covers the entire eastern side of Stromboli Island (Fig. 2d,e,f). The deposit mass values (sample weight on the sampling area in g/m^2^; Supplementary Information 2 Table S1) range between 18.31 and 145.38 g/m^2^, displaying a scattered distribution across the field (Fig. 2a) and no relation to the distance from the source. The highest mass values were recorded on the beach of *Scari* (STR04), close to the helipad (Fig. 2a), at *Campo sportivo* (STR06) and on the right rim of the SdF (STR08). Lower mass values were recorded for the samples within the village of Stromboli, such as STR05 and STR07, and STR01—collected on the road that climbs from *Scari* to *San Vincenzo*—is by far the least abundant.

**Figure 3:**
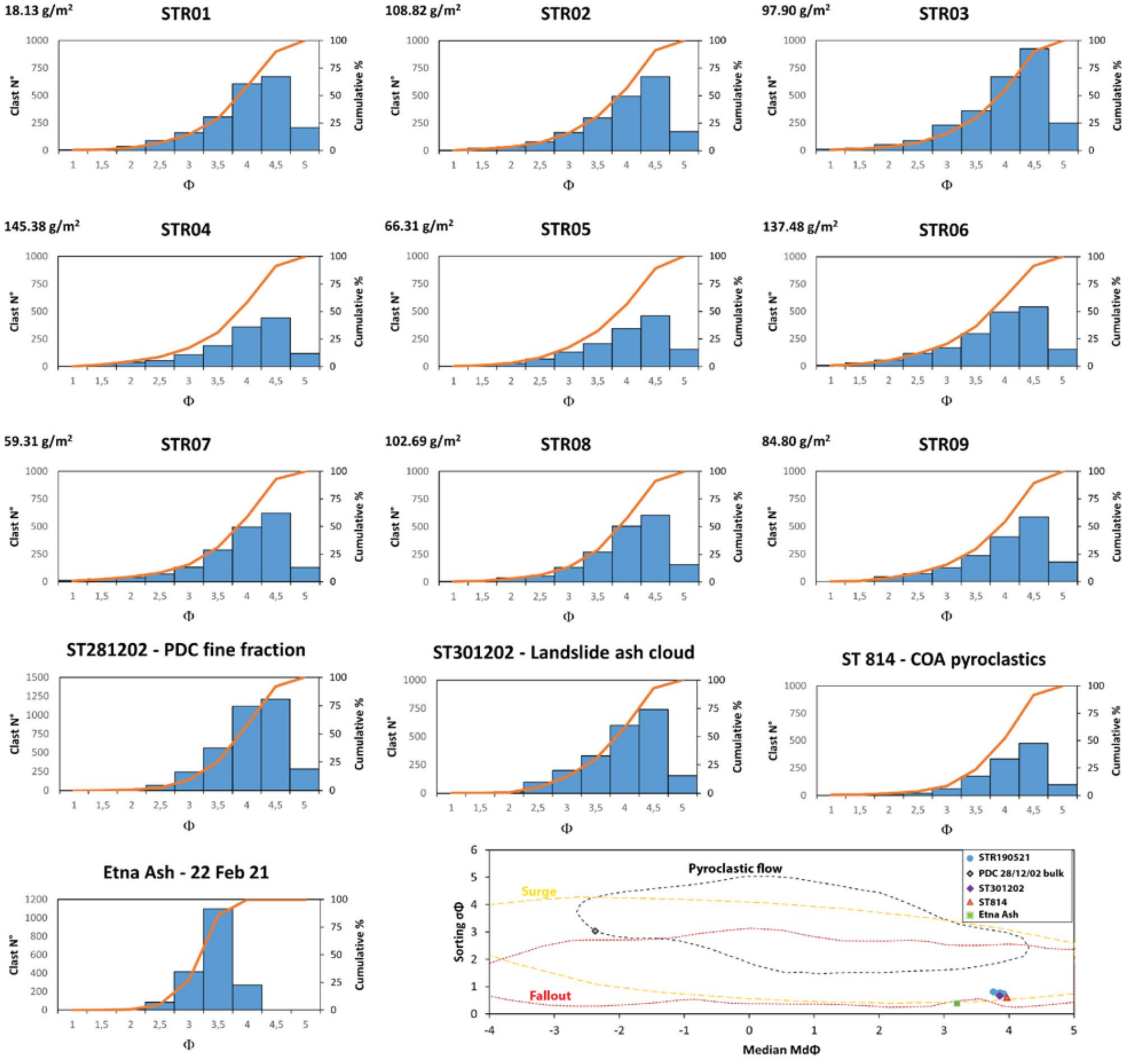
Plots illustrating the grain size distribution histograms and the cumulative curves of the ash samples. Note that ST281202 refers to the ash fraction of the PDC deposit. The binary plot median vs. sorting (modified after^27,28^) denotes the fields of fallout and PDC (either flow or surge) deposits. Note that the PDC28/12/02 refers to the bulk sample.

All the samples are characterised by a great abundance of clasts well below the lower GSD limit of 5ϕ. Within the analysed size interval (Fig. 3), the 80–85% of the sample is in the range between 3.5ϕ and 5ϕ, with 55–60% included between the 4ϕ and 4.5ϕ range. GSD histograms are very similar to one another, displaying a unimodal trend with peaks at 4.5ϕ and 4ϕ (Fig. 3; Supplementary Information 2 Table S1). Md_ϕ_ ranges between 3.76 and 3.92, and sorting is overall good (σ_ϕ_ between 0.72 and 0.81).

The samples of the 2002 eruption (ST281202 and ST301202) and the COA ash tuff (ST814) display very similar GSD curves, revealing comparable size abundance (Fig. 3). By contrast, Etna Ash sample has Gaussian (normal) GSD (Fig. 3), with a peak at 3.5ϕ that contains about 60% of the sample, having lower median (Md_ϕ_ = 3.20) and greater sorting (σ_ϕ_ = 0.39). In the Md_ϕ_ vs σ_ϕ_ diagram (Fig. 3; after Walker^27^), all the samples cluster in a very close space within the field of fallout/surge, except the Etna Ash, which is separated but still in the same field, and the bulk sample of the 28 December 2002 PDC, which plots in the field of pyroclastic flows.

### Clast shape analysis

**Figures 4:**
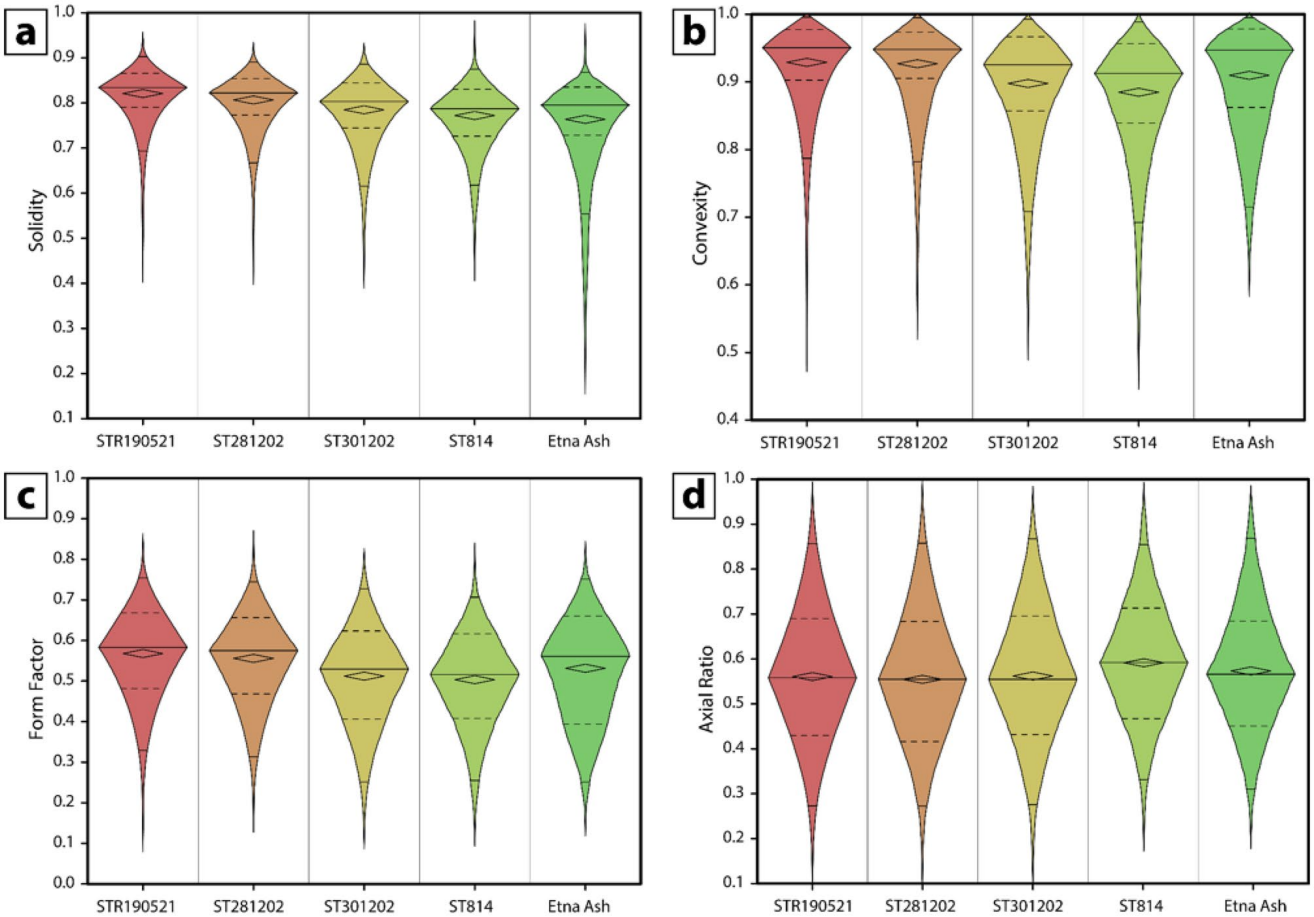
Box plots (**a**),(**b**),(**c**),(**d**) illustrate the range of solidity, convexity, form factor and axial ratio of the ash clasts. Solidity and convexity have higher values for samples STR190521, ST281202 and ST301202 revealing that the majority of the clasts have equiaxial smooth shapes. Samples ST814 and Etna Ash have lower values of the same parameters, indicating clasts with much highly complex and elongated shapes.

Results of clast shape analysis are shown in Fig. 4 (Supplementary Information 2 Table S1). Among the large number of the shape descriptors, we focused on those parameters that are related to fragmentation, transport and settling, recording effects of clast-to-clast interaction. In particular, we report some morphological parameters suggested by Liu et al.^29^, namely solidity (SLD; a measure of morphological roughness, sensitive to large-scale concavities), convexity (CVX; a measure of textural roughness, sensitive to small-scale concavities or protrusions), form factor (FF; a measure of the deviation of a particle from a circle, either changing particle elongation or increasing surface roughness), and axial ratio (AR; sensitive to particle elongation). High values of convexity and solidity (> 0.9) characterize equiaxial rounded particles with smooth, straight-edged outlines, which might indicate the result of comminution processes and surface abrasion. Vesicular ash particles with complex and elongated shapes share lower values (< 0.8) of convexity and solidity^29,30^.

Samples of the 19 May 2021 ash clouds (STR01-09) have very similar shape parameters to one another and are therefore plotted as a whole (STR190521). Overall, all the samples display a range of solidity, from very low to moderate, in a narrower interval of convexity, from moderate to high^30^. Samples of the 19 May 2021 share moderate values of solidity (50% of the sample being between quartile 1, Q1 = 0.79, and quartile 3, Q3 = 0.86, with Median = 0.83) with high values of convexity (Q1 = 0.90, Q3 = 0.97 and Median = 0.95). The 28 December 2002 PDC ash (ST281202) has low values of solidity (Q1 = 0.77, Q3 = 0.85 and Median = 0.82) associated with high values of convexity (Q1 = 0.92, Q3 = 0.97 and Median = 0.94). The 30 December 2002 ash-cloud (ST301202) has low values of solidity as well (Q1 = 0.74, Q3 = 0.84 and Median = 0.80), but with convexity ranging from moderate to high (Q1 = 0.85, Q3 = 0.92 and Median = 0.96). The COA ash tuff (ST814) has very low values of solidity (Q1 = 0.72, Q3 = 0.83 and Median = 0.79) and convexity ranging from moderate to high (Q1 = 0.84, Q3 = 0.96 and Median = 0.91), similarly to the Etna Ash sample, with solidity ranging between Q1 = 0.72 and Q3 = 0.83 (Median = 0.79) and convexity between Q1 = 0.86 and Q3 = 0.98 (Median = 0.94). Also, Etna Ash is the sample that reaches the lowest values of solidity, representative of the most concave forms^29^. Values of form factors and axial ratios are in a very narrow range and there are no differences among the samples. These values suggest a great abundance of sub-equant particles.

### Component analysis

The 19 May ash cloud deposits comprise mainly lava fragments, glassy vesicular fragments and volcanic shards, and free crystals.

**Figure 5:**
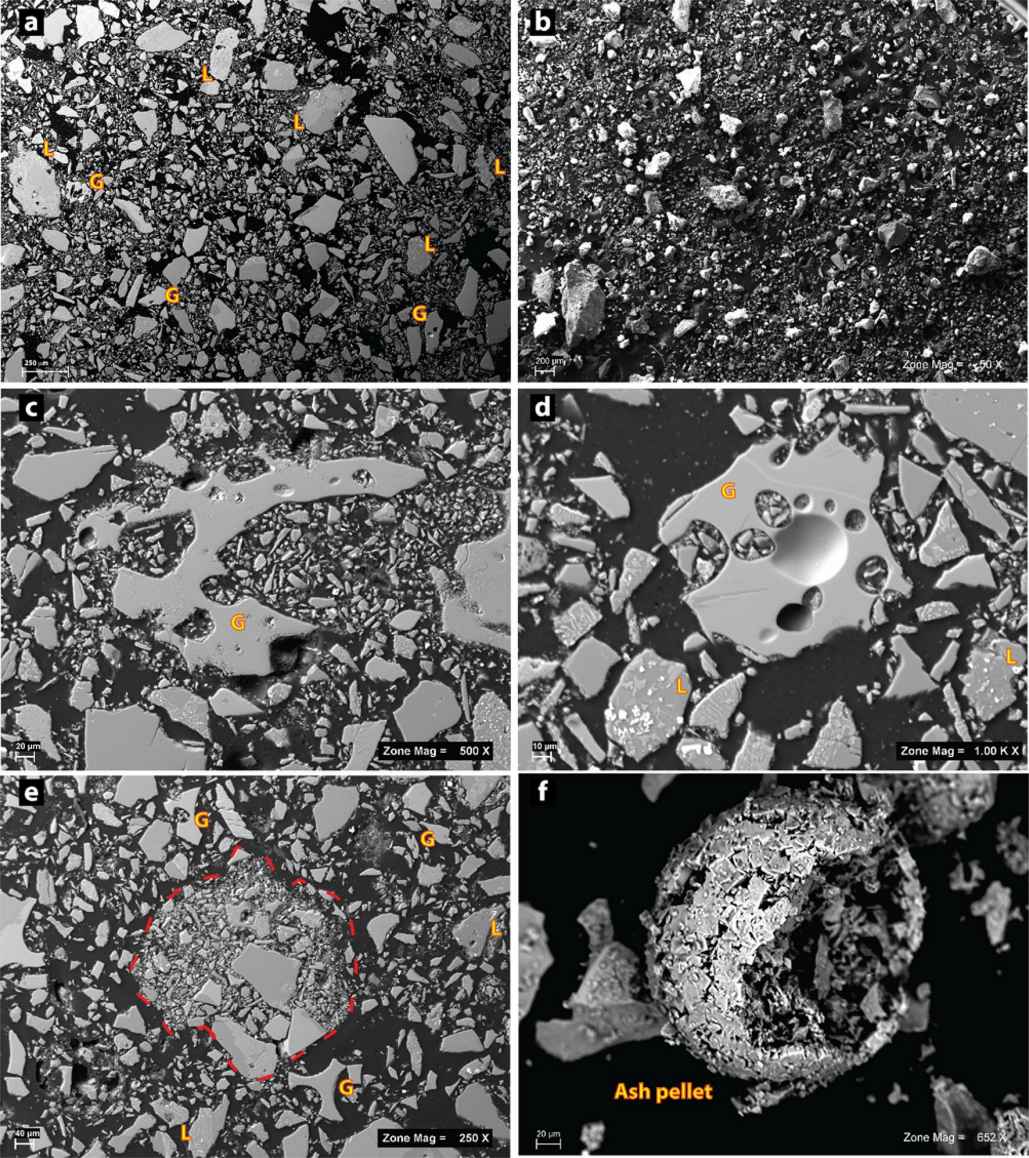
SEM photographs illustrating the textures of the deposits. Photos (**a**) and (**b**) are the general views of the 19 May 2021 ash cloud deposit in 2D and 3D respectively. The majority of the clasts are lavas (L) and there are only a few glassy clasts (**G**). (**c**) and (**d**) are close ups of two glassy clasts with LP composition (see section "Glass composition"). The clast in (**c**) reveals a inhomogeneous texture with portions of clear unaltered groundmass coexisting with portions with abundant plumose crystallites, a symptom of recycling. The glassy clast in figure (**d**) has a clean unaltered groundmass, with few microlites of plagioclase. (**e**) and (**f**) illustrate two ash pellets in 2D and 3D respectively.

Lava fragments constitute about 60% of the samples and consist of dense clasts displaying variable microlite contents in a cryptocrystalline groundmass (Fig. 5a,b). Glassy shards represent about 15% of the samples, are up to 350 μm, and comprise equant to slightly elongated fragments, dense to moderately vesicular with spaced and non-collapsed bubbles (Fig. 5c,d). Glassy fragments are generally aphyric with rare microlites of (swallow-tailed) plagioclase, but some contain rare micro-phenocrysts of plagioclase, clinopyroxene and olivine (Fig. 5d).

Clasts are often reddish to yellowish in color with an incipient alteration patina. Alteration of glassy clasts is also visible in the BSE image, since they reveal inhomogeneous crystallinity of the groundmass with portions of fine-grained plumose crystallites coexisting with glassy areas (Fig. 5c).

Free crystals are also abundant (about 25% of the samples) and comprise plagioclase, clinopyroxene and olivine. Ash clasts aggregate into ash pellets up to 500 μm in diameter with a fairly regular rounded outline (Fig. 5e,f). 3D imaging of ash pellets reveals the presence of sodium chloride (NaCl) crystals on their surface (Fig. 5f), suggesting the involvement of seawater steam in their formation.

Samples studied for comparison, such as the fine ash related to the 2002 PDC (ST281202) and landslide (ST301202), have a very similar componentry with 65% of lava and altered volcanic rocks, 16% of glassy vesicular fragments and volcanic shards, and 18% of loose crystals.

### Glass composition

**Figure 6:**
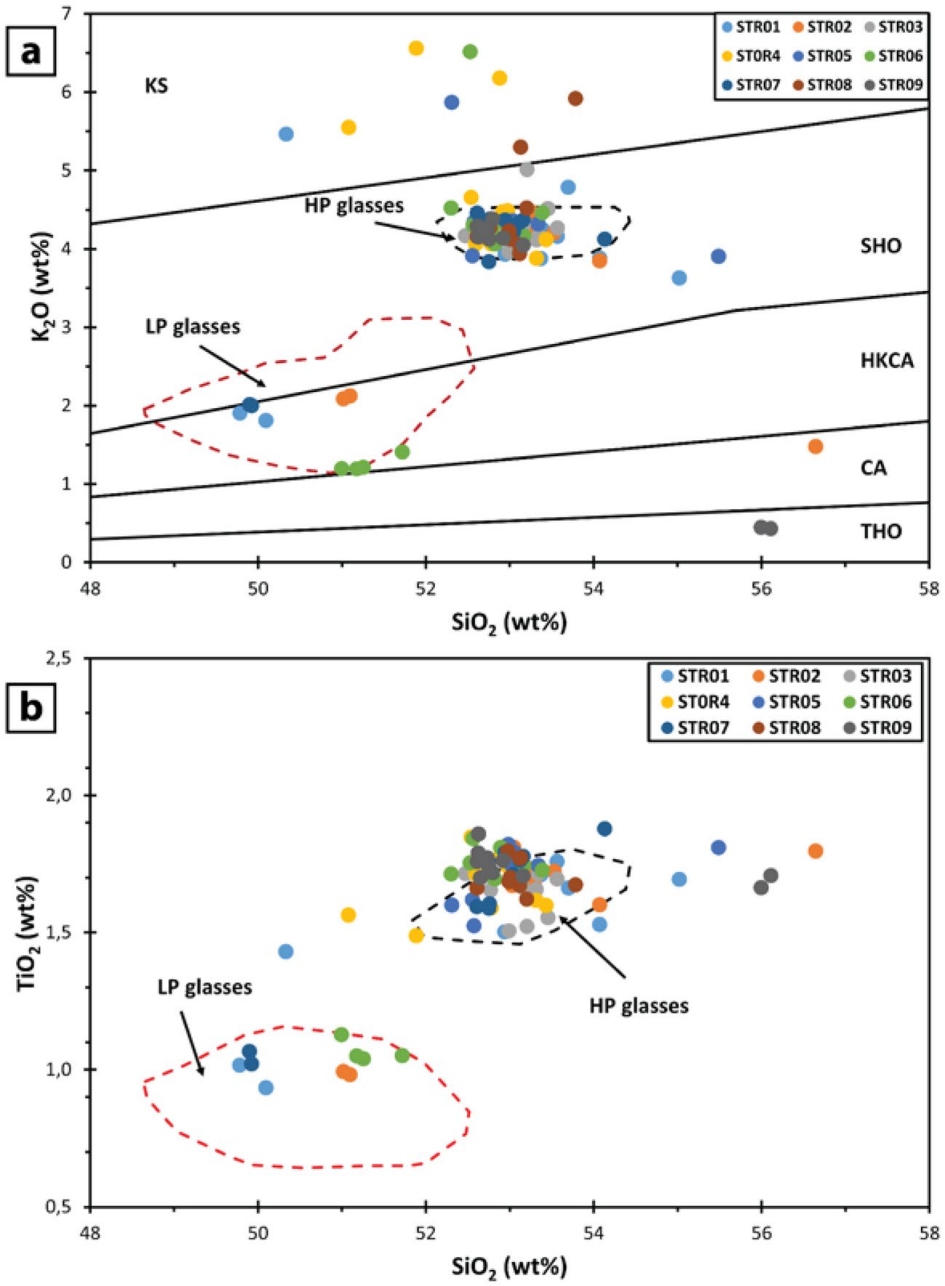
Plots of geochemical composition on groundmass glass. (**a**) is the SiO_2_ vs K_2_O plot (modified after Peccerillo and Taylor^31^). Compositional fields defined with the dashed lines are from Pichavant et al.^39^ and represent the compositions of 2019 paroxysms and of typical products erupted at Stromboli during the ordinary (HP; High Porphyricity) and paroxysmal (LP; Low Porphyricity) activities. (**b**) represents the SiO_2_ vs TiO_2_ plot showing that silica-rich and low-alkali groundmass in (**a**) have the same TiO_2_ composition of the HP glasses.

Major element compositions of glass have been acquired on vesicular glass fragments and glass shards (10 clasts each sample; Supplementary Information 3 Table S2), apparently showing no evidence of chemical alteration and in pristine shape. Samples have a rather similar composition and range from High-K calc-alkaline basalt to shoshonite on the SiO_2_ vs K_2_O diagram (Fig. 6a; after Peccerillo and Taylor^31^). Four groups of glass compositions can be distinguished. The more populated group has SiO_2_ ranging between 52.30–55.49 wt% and K_2_O between 3.63 and 4.79 wt%, and clusters within the field of high-porphyricity glasses (HP)^32,33^, typically erupted by the current Strombolian activity^32–38^, and during paroxysmal explosions including the recent ones (3 July and 28 August 2019)^39^.

The second group includes few clasts, with SiO_2_ ranging between 49.78 and 51.72 wt% and K_2_O between 1.19–2.01 wt%, plotting within the field of low-porphyricity compositions (LP), erupted during paroxysmal (and perhaps major) explosions in association with the HP scoria^37–43^. The other two groups of glasses are more scattered. The third one shows a wide range of SiO_2_ (between 50.34 and 53.79 wt%), and higher K_2_O content than the HP compositions (K_2_O = 5.30–6.56 wt%). These compositions display a good affinity with the potassic alkaline series (KS) that erupted during the Neostromboli period^44,45^. Lastly, the fourth group includes a few clasts that plots at an average SiO_2_ wt% of 56.3 but shows a very low alkali content (average Na_2_O + K_2_O = 0.96 wt%). The binary plot (Fig. 6b) SiO_2_ *versus* an immobile and incompatible element like TiO_2_ shows that the third and the fourth groups have a comparable concentration of this element to HP glasses.

## Discussion

**Figure 7:**
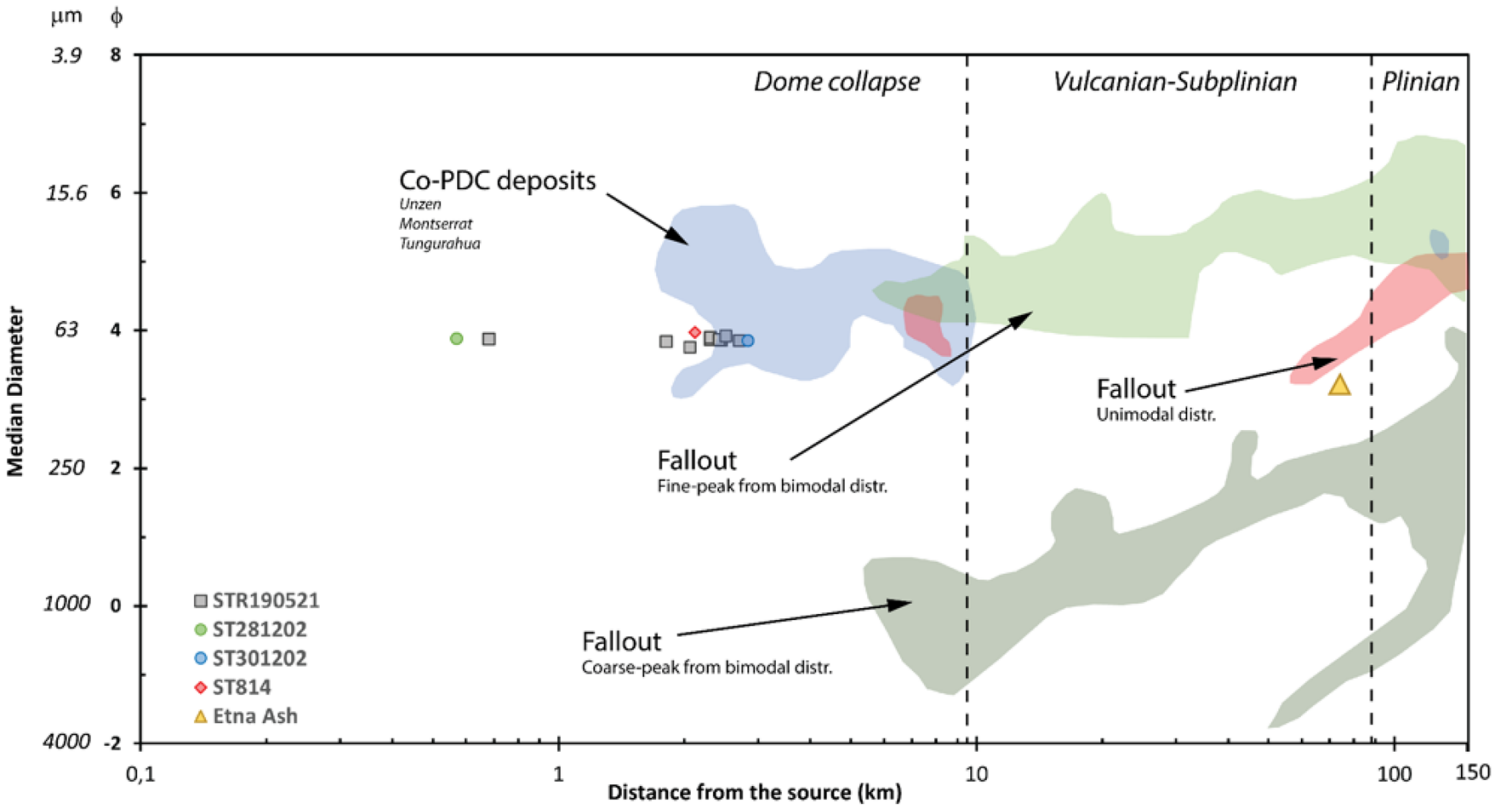
Variation of grain size (Mdϕ) with the distance (km) from the vent, modified from Engwell and Eychenne^46^. Fields of co-PDC (in blue) and fallout (pale green—fine peak from bimodal distribution; dark green—coarse peak from bimodal distribution; red—unimodal distribution) deposits are plotted. Vertical dashed lines roughly approximate the general trend of the deposit dispersal as a function of the eruptive style.

The ground distribution of the 19 May 2021 ash cloud deposit is limited to the NE sector of the island, thus is mainly controlled by the cloud dispersal due to the wind direction and the PDC path. The source of the ash cloud is ascribed to the SdF, the portion of the NW flank affected by the PDC. Neither the mass distributions on the ground nor the GSD are correlated with the distance from the source, which is expected for an ash fall deposited by an eruptive column. The scattered ground distribution can be related to a non- homogeneous mass load within the ash cloud (see Fig. 1e, f and movie in Supplementary Video 1) and local turbulence effects within the village (e.g., building walls and narrow streets). The GSD of the 19 May 2021 ash samples is notably skewed toward fine sizes. This feature is shared with the ash samples of the beginning of the 2002–2003 eruption, as well with the COA ash tuff. By contrast, the GSD of the distal Etna Ash fallout reveals different characteristics, being slightly coarser and better sorted. Also, GSD of the 19 May 2021 ash cloud compares well with the ash cloud elutriated from the 2014 basaltic PDC of Etna (Unit 1, FC5)^8^, despite extending towards coarser dimensions. Figure 7 (modified from Engwell and Eychenne^46^) displays the distance from the source *versus* the median diameter, revealing that, despite differences in chemistry of the magma and eruptive styles, the studied ash cloud samples are similar to other worldwide co-PDC deposits. The consistency of GSD, even at more proximal locations, is in agreement with the indication that particle entrainment in co-PDC plumes are size-selective processes^46^.

The componentry of the 2021 ash cloud deposits reflects the predominance of lava and scoria fragments and loose crystals. These components derive from the material forming the collapsed crater rim and/or from the volcaniclastic deposits forming the infill of the SdF that were ingested by the PDC along its path. It is not possible to distinguish and quantify these two contributions. Similar componentry abundance has been observed for the 2002 samples of Stromboli, and also for the 2014 PDC-generated ash cloud of Etna^8^ where blocky particles are predominant on spongy and fluidal ones.

Vesicular glassy fragments and volcanic shards have subordinate abundance, and the majority of clasts reflect the composition of the HP magma feeding the ordinary Strombolian activity including the most recent ones (Fig. 6). The presence of a few clasts showing the composition of LP magma is not clear. LP compositions have been predominantly observed in paroxysms and the only exceptions pertain to the ash produced by discrete explosions occurring in March 2003, when the shallow plumbing system was reactivated after the crater collapse at the end of December 2002^47^, and to the highly vesicular ash fragments erupted during normal strombolian explosions in September 2008^48^. Taking into account that no significant explosive activity was observed during the formation of the PDC, we interpret the LP clast as accidental lithics assimilated during the transport along the SdF, probably in the medial portion according to^49^.

Similarly, silica-rich compositions have been produced in the past during the activity of Vancori (26–13 ka) and Neostromboli (12–4 ka) volcanoes; however, these rocks show alkali concentrations ranging from of HCKA and Potassic series ^45,50^. Thus, these high-silica and low-alkali glasses (Fig. 6) cannot be related to some known composition erupted from Stromboli in the past, but are attributable to the alteration of the fine material accumulated within and outside the crater, and successively remobilized by the collapse. Possible mechanisms of alteration include the hot gases and the recycling within the craters^51,52^ The presence of apparently-fresh glass shards, whose lithic nature can be recognized only on the basis of compositional information, would suggest considerable caution when using ash in the context of petrological monitoring^53,54^.

Lastly, a distinctive feature of the deposit is the aggregation of ash into pellets due to the abundance of steam within the plume, produced by the interaction of the hot PDC material with the sea water. Similarly, from 30 December 2002, after the partial SdF collapse, the interaction of the hot material (collapsing lava fronts and associated landslides) with the sea, produced a copious fall out of ash pellets over the whole island lasting several days (personal observation of M.P.).

The shape of the clasts shows slight differences among the analysed samples. Samples of the 19 May 2021 ash cloud have high values of solidity and convexity, typical of smooth equiaxial clasts, and are comparable to those of the 2002–2003 eruption and to the 2014 PDC-generated Etna ash cloud (FC5)^8^. By contrast, the COA ash tuff and the distal Etna Ash fallout reveal lower values of solidity sharing similar values of convexity, suggesting the presence of a population of clasts with more complex and elongated shapes. In this regard, we should keep in mind that some shape parameters are very sensitive to pixel resolution compared to others^55^. In particular, parameters such as area and length, that are found on unambiguous pixels within grains, are much less sensitive compared to perimeters, which is calculated from the edge pixels that display smoother or rougher outlines as a function of the set resolution^55^. For this reason, area and length-based parameters, such as solidity and axial ratio, are more reliable than perimeter-based ones, such as convexity and form factor.

Clast shape analysis provides, in principle, information about fragmentation and transport processes^1,29,30,56–60^.

**Figure 8:**
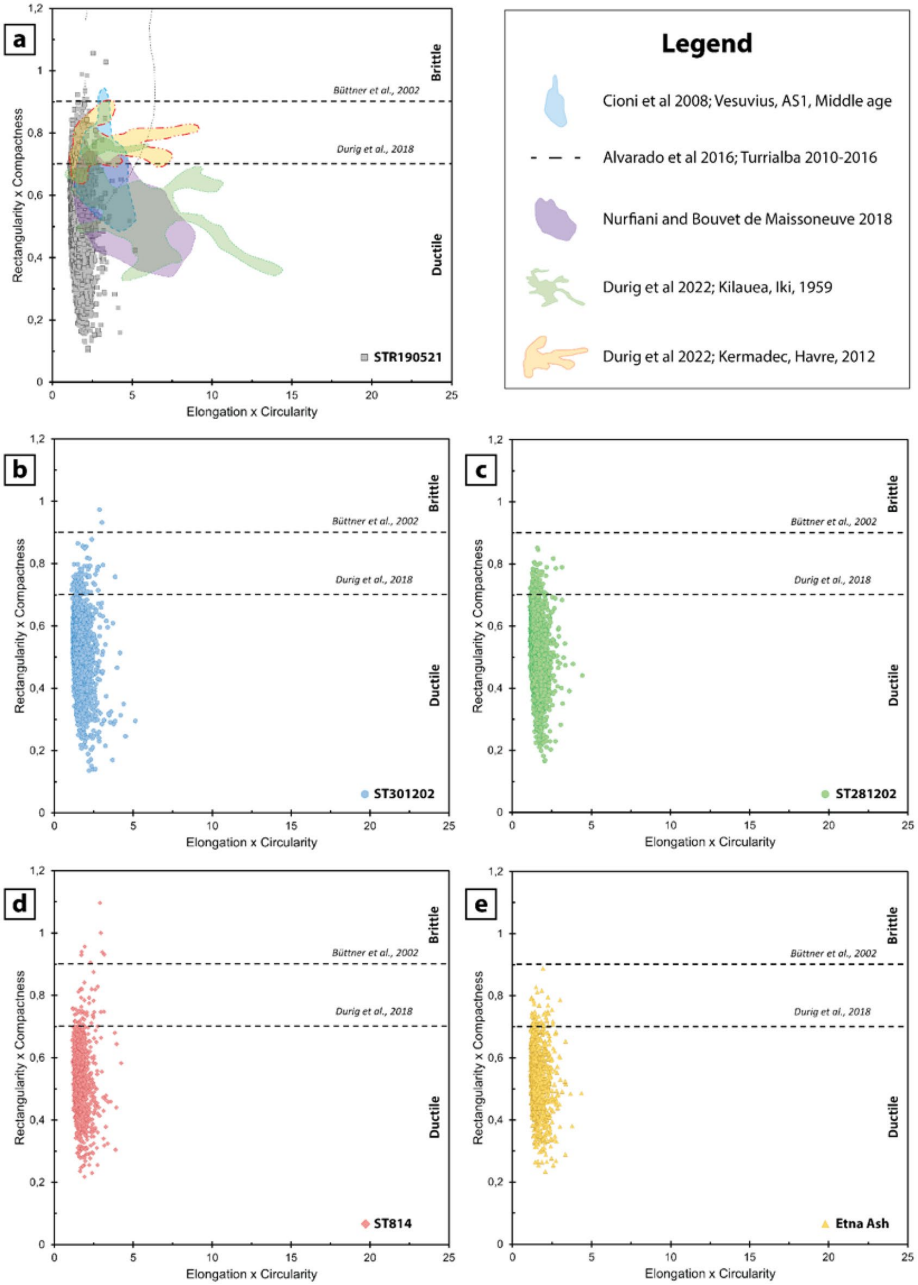
Elongation × Circularity versus Rectangularity × Compactness plot, modified after^61^, aim to identify the fragmentation style by means of particle shapes. Two thresholds, suggested by Büttner et al.^61^ and Dürig et al.^63^ are shown as dashed horizontal lines, separating the field of particles generated by brittle fragmentation (at the top), from those produced by ductile fragmentation (at the bottom). Each plot shows one individual sample, and the fields of some literature samples, reported in the legend, are annotated.

Büttner et al. ^61^ designed a classification diagram based on a combination of shape parameters, for instance elongation per circularity versus rectangularity per compactness, to distinguish between brittle and ductile styles of fragmentation. In these plots (Fig. 8) all the samples display very low values of elongation per circularity while extending at a greater interval of rectangularity per compactness. The majority of the clasts of the 2021 ash cloud and 2002 deposits (Fig. 8a,b,c) plot within the field of ductile fragmentation, with only a minor amount extending above the threshold of brittle fragmentation. COA ash tuff (Fig. 8d) reveals almost exclusively a brittle fragmentation origin, similarly to the Etna Ash (Fig. 8e), which is the distal deposit of a lava fountain.

**Figure 9:**
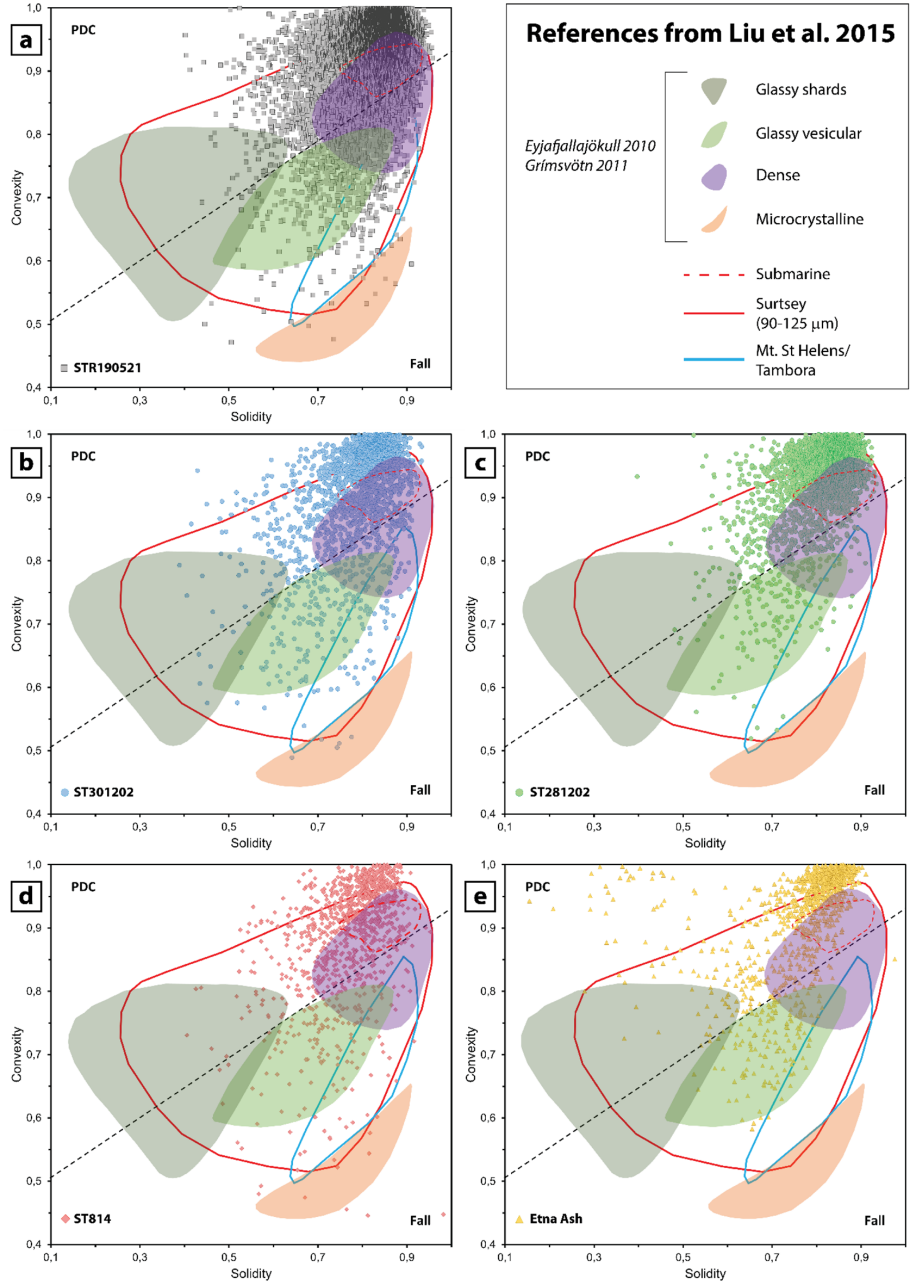
Solidity versus convexity plots (after^29^). The black dashed line marked the threshold between flow (PDC) deposits and fallout. Each plot shows one individual sample, and the fields of some literature samples, reported in the legend, are annotated.

To investigate transport effects on the clast shape, we used the solidity versus convexity diagram (Fig. 9), proposed by Liu et al.^29^), that distinguishes between flow (PDC) deposits, which are characterised by high convexity (0.7 < CVX < 0.9) and fallout deposits (0.5 < CVX < 0.8). All the samples analysed in this study (Fig. 9) plot in a wider range of solidity (0.15 ≤ SLD ≤ 0.97) compared to convexity (0.45 ≤ CVX ≤ 0.99). The majority of the clasts cluster within the PDC field, although all samples have some clasts within the field of fallout. Samples related to the 19 May 2021 ash clouds, the 2002 ash deposits and the COA ash tuff (Fig. 9a,b,c,d) have a predominance of clasts within the field of dense components, compared to the distal Etna Ash fallout (Fig. 9e). Etna Ash shows the main difference extending toward lower solidity values while sharing comparable convexity (Fig. 9e).

A predominant secondary brittle fragmentation, due to the comminution of clasts during the transport, is expected for the ash cloud related to the PDC and landslides at Stromboli. As outlined before, the ash cloud mostly ingested the loose detritus accumulated on the Stromboli crater terrace and SdF slopes, which consist of vesicular glassy ash and lapilli produced by the ductile fragmentation of the normal strombolian activity and dense crystalline clasts from the crumbling of lava flows. Abrasion experiments reveal that vesicular clasts are more susceptible to splitting, generating more fine ash with irregular shapes, compared to crystalline components, which are more prone to rounding^62^. Therefore, the great amount of fine ash can be ascribed to clasts comminution processes, although the prevalence of dense crystalline components records an overall equiaxial shape, revealing a paucity of elongated clast with complex morphology.

Finally, we wish to make some considerations on the recurrence, in the stratigraphic record of Stromboli, of fine ash deposits, similar to that investigated here. Discontinuous, pinkish fine ash layers have been described in the stratigraphic record of Stromboli^64,65^ and ash fall phenomena are also reported within the historical chronicles without a clear association with some explosive events^62,66^ The fine ash deposition, similar to that occurring on 19 May 2021, would have been completely overlooked in the past without the availability of the present-day monitoring network and cameras. Moreover, these deposits are ephemeral since they are susceptible to fast washout by rain, remobilization by wind and rapid erosion, therefore they are difficult to be recognized or sampled in natural outcrops or in stratigraphic trenches.

Using a reasonable proportionality principle, if we consider that the 19 May 2021 PDC, generated from the collapse of a mass < 10^4^ m^3^, produced only a 1 mm thick ash deposit, we could argue that pinkish/reddish ash layers found within the stratigraphic sequences, with thickness up to several cm (such as the T2c of^64^, which is 5–6 cm thick), excluding the likely loss of a reworked portion, would have been generated by events some orders of magnitude greater, or by a series of repeated collapses close in time. Considering that many volcanoes worldwide are capable of producing PDCs, and in some cases tsunami waves (e.g., Pacaya, Guatemala^67^; Sangay, Ecuador^68^; Kambalani, Kamtchatka^69^ 2006; Fuji, Japan^70^; Ritter Island, Papua New Guinea^71^; Anak Krakatau, Indonesia^72^) the prompt recognition of ash layers that correlate with this kind of phenomena would have a great impact on hazard assessment.

## Conclusions

On 19 May 2021 an ash cloud was produced at Stromboli from the PDC generated by the gravitational collapse of a relatively small portion (10^4^ m^3^) of the crater terrace, which emplaced a very thin, continuous ash layer highly susceptible to reworking. This kind of the activity has been observed by the monitoring networks and, for the first time, we have been able to collect the primary ash cloud deposit soon after its deposition.

The comprehensive characterization of this deposit compared to 2002 ash samples that relates to a tsunamigenic collapses of SdF reveals several similarities. All of these deposits have a consistent grain size distribution with a great abundance of fine ash and all of them can be ascribed to co-PDC deposits. On the other hand, Etna Ash sample that is related to an explosive event displays different granulometric features typical of fallout deposits.

Concerning component abundance, the 19 May 2021 and 2002 ash layers are formed by predominantly lava fragments and subordinate glassy fragments, in agreement with the mechanisms of formation of the deposit (gravitative PDC). Since no significant explosive activity was observed during the formation of the PDC, vesicular glassy fragments which reflect both composition of HP and LP magmas has not an undisputable juvenile origin, but likely derived from remobilized material of SdF infill.

Finally, clast morphology provides indication on the fragmentation and transport processes, in particular ash particles of 19 May 2021 and 2002 eruption are remobilized and comminuted during the PDC, whereas the COA ash tuff and the distal Etna Ash have a portion of particles showing pristine shapes due to magmatic fragmentation.

The volcanological characterization of the 19 May 2021 ash cloud layer aims to discriminate among deposits that look similar in the field but might be related to a large range of diverse eruptive phenomena, in order to create a benchmark useful to compare similar types of deposits worldwide. Lastly, we would like to stress the importance of fine pinkish ash tuff within volcano’s stratigraphic records, as they could be related to processes of edifice disruption that will make differences in the perception of volcanic hazard.

## Methods

From 16.30 CET (ca. three hours after the event of collapse), nine ash samples were collected on clean flat surfaces, along a SE-NW traverse across the Stromboli village, downwind from the crater area at variable elevation from 0 to 150 m a.s.l. (Fig. 2a; blue stars). Due to Civil Defence safety limitations, it was not possible to verify whether the ash cloud deposit occurred at higher elevations. On 20 May, the day after the event, a survey was conducted on the western side of the island, from *Ginostra* to *Punta dei Corvi*, assessing that the deposit had not emplaced in this area. The sampling was performed using a brush and a dustpan, and for each sample the sampling area was measured in the field to calculate the mass (g/m^2^; annotated in Fig. 2a and Supplementary Information 2 Table S1).

For comparison with the 19 May 2021 Stromboli PDC ash, we also studied four other fine ash deposits, three of them from Stromboli and one from Etna. Sample 1 (ST281202; Fig. 2a) is the ash fraction of the 28 December 2002 PDC, emplaced at the *Spiaggia dei Gabbiani*. This PDC was produced by the gravitational collapse of a thick spatter deposit accumulated over the steep slope during an explosive fountaining activity from a lateral vent at ~ 650 m asl within the SdF (Pioli et al., 2008). Sample 2 (ST301202; Fig. 2a), which was collected at Punta Lena, is the cumulative ash deposit emplaced during the major 30 December 2002 landslide and the minor landslides occurring in the following days (with a total collapsed volume of at least 10^7^ m^3^ of the SdF infill, for a thickness of at least 65 m)^25^. Sample 3 (ST814; Fig. 2a,c; Unit C7 from Porreca et al. ^73^) is a pink ash tuff sampled at the top of the pyroclastic succession exposed just below COA, which relates to a Holocene phreatomagmatic eruption at the end of the Neostromboli cycle. Porreca et al.^73^ interpreted this unit as the fine ash fallout occurring during a relatively short pause after the emplacement of the underneath pyroclastic density current; instead, we consider this ash tuff as the co-PDC ash cloud deposit (Fig. 2c). Sample 4 (Etna Ash; Fig. 2b) is the distal ash fallout of the 22 February 2021 paroxysm of Etna. It resulted from a 50 min-long lava fountain, with jets of magma about 1 km high, that produced an eruptive column of about 10 km above the volcano summit with dispersion W-NW (INGV-OE Report of 02 March 2021), and was collected at about 75 km NW from the Etna summit craters (in the town of San Mauro Castelverde, Sicily).

Bulk samples were mounted in epoxy resin and polished for componentry, textural and geochemical analyses, following the procedures described by Ross et al.^74^. These analyses were performed at the Istituto Nazionale di Geofisica e Vulcanologia, Sezione di Pisa (INGV-Pisa) by using a scanning electron microscope (SEM) Zeiss EVO MA. Sample ST281202 was first sieved using a 2ϕ mesh sieve (250 microns; Phi size = − log2(diameter mm)), whereas the GSD statistics of the bulk sample were provided from Solano ^75^. GSD, clast shape analysis and componentry were carried out with image analysis techniques, on a mosaic of 2048*1536 pixel pictures acquired at a magnification of 200× and using the ImageJ software (http://imagej.nih.gov/ij/).

Images were thresholded to binary and filtered to eliminate outliers with a double erosion operation followed by a double dilation one. This filtering combination, whilst preserving the original particle shape, minimised the artificial complexity added to the particle outline during thresholding and prevented the effect of particles overlap, which could otherwise represent a consistent source of error given the very fine nature of the ash and the presence of aggregates. Hand corrections were also performed where needed.

Measurements were performed on particles that do not intersect the picture edge with the macro routine developed by Liu et al.^29^, and include: area (including internal holes), perimeter, major and minor axes of the best-fit ellipse, width and height of the bounding rectangle, maximum and minimum Feret diameters, and convex hull area and perimeter.

The GSDs are calculated, with an interval of 0.5ϕ, using the maximum Feret diameters, on clasts with a surface larger than 350 μm^2^, equivalent to a sphere with a diameter of ca. 22 μm. Therefore, the GSD lower limit is arbitrarily set at 5ϕ (< 32 μm). Similarly, shape parameters are considered for clasts with a pixel density higher than 750, as suggested by Liu et al. ^29^. Given that the pixel size of the acquired images is 0.68947 μm, the section of a cubic particle of 750 pixels area has a diagonal of ca. 27 μm, having a size comparable with the previous method. By the use of these thresholds, the number of analysed particles range between 1300 and 3500 per sample, ensuring that shape parameters converge to stable values (relative standard deviation < 0.2) for the examined grains size^30^.

Modal analysis of components has been performed on five samples, three from the 19 May 2021 ash cloud (STR01, 04 and 08) and two from the December 2002 eruption (ST281202 and ST301202), by counting between 400 and 500 clasts per sample on SEM backscattered mosaic of pictures.

Major elements geochemical analyses were performed at the INGV-Pisa using a Zeiss EVO MA SEM equipped with an Oxford energy dispersive X-ray detector (EDS). Operative conditions were accelerating voltage 20 kV, beam current ≈2nA, working distance 8.5 mm. The VG-2 basaltic glass secondary standard was repeatedly analysed during the EDS acquisitions to test the accuracy of the data.

## Supplementary Information

Supplementary Information 1.

Supplementary Video 1.

Supplementary Information 2.

Supplementary Information 3.

## Data Availability

All data generated or analysed during this study are included in this published article and its supplementary information files.
