# Supplementary material for: Physical and morphological characterization of the 19 May 2021 ash cloud deposit at Stromboli (Italy)
Source: Sci Rep. 2022 Jun 24;12:10777. doi: 10.1038/s41598-022-14908-1 (PMC9232538; doi:10.1038/s41598-022-14908-1)
Supplement: Supplementary file 1 — Supplementary Information 1. [file 41598_2022_14908_MOESM1_ESM.docx]

Supplementary video 1 : Video of the PDC and related ash cloud spreading on Stromboli village taken by a tourist (courtesy of Milena Marchioni – Viaggi&Vulcani) from the Ficogrande beach.
